# Supplementary material for: Are carbon emissions trading and green financial instruments synergistic? -Comprehensive quantitative research based on content analysis
Source: PLoS One. 2024 Mar 7;19(3):e0298601. doi: 10.1371/journal.pone.0298601 (PMC10919601; doi:10.1371/journal.pone.0298601)
Supplement: S1 Data — (ZIP) [file pone.0298601.s001.zip › Data/地级市Z_F工作报告.zip!/░▓╗╒/┘±╓▌╩╨/└┤╫╘╣1⁄2╓┌║┼í╛┬φ┐╦╩2╛▌═°í┐_2018.docx]

各位代表：
　　现在，我代表市人民政府，向大会报告政府工作，请予审议，并请市政协委员和其他列席人员提出意见。

****一、2017年工作回顾****

　　2017年是本届政府任期的第一年。我们在省委、省政府和市委的坚强领导下，紧紧依靠全市人民，全面贯彻落实党的十八大和十九大精神，以习近平新时代中国特色社会主义思想为指导，坚持稳中求进、优中求快、奋发有为工作总基调，按照“深入践行五大发展理念，奋力走在皖北振兴前列”工作总要求，坚持“四个导向”，紧盯“四个点问题”，深入实施五大发展行动计划，着力提高发展质量和效益，全面做好改革发展稳定各项工作，较好地完成了市四届人大一次会议确定的各项目标任务。
　　——经济实力持续增强。全市地区生产总值1184亿元，增长9.2%、居全省第一位、连续三年超过全省平均水平；财政收入171亿元，增长16%、居全省第三位，总量赶超两个市、居全省第十位；固定资产投资1067亿元，增长22%、居全省第二位；社会消费品零售总额551亿元，增长11.9%，主要经济指标增速继续保持全省前列。
　　——产业结构持续优化。主导产业加快升级，新兴服务业快速发展，三次产业比由2016年的19.7：38.7：41.6优化为17.7：40.1：42.2。规模工业企业达1032家，规模工业增加值增长10.4%、创近两年新高、居全省第一位。医药制造业、战略性新兴产业、农产品加工业产值分别增长25%、23%、15%。
　　——发展后劲持续提升。实施省市重点项目542个、完成投资780亿元，26个10亿元以上重大项目开工建设，128个亿元项目竣工投产或主体完工，省重点项目投资完成率、开工率、竣工率均居全省前三位。商合杭高铁亳州段墩柱全面完工、高铁南站站房即将开工，亳州机场预可研文件已报国家待批，引江济淮亳州段工程全面开工，亳蒙高速立项获批，亳蚌城际铁路前期工作加快推进，137公里国省干线公路改建及路面改善工程顺利完工。
　　——幸福指数持续攀升。脱贫攻坚再战再捷，89个村成功出列，137341人稳定脱贫，谯城区、蒙城县被列入全省首批4个拟脱贫摘帽县区。居民人均可支配收入增长9.8%、农村常住居民人均可支配收入增长9.6%，均居全省第二位；城镇常住居民人均可支配收入增长8.8%，居全省第四位。32项民生工程全部完成年度任务，总投资144.2亿元。改造农村危房3万户，建设农村道路畅通工程2143公里，解决58.4万农村居民安全饮水问题。平安亳州建设扎实推进，人民群众的安全感和满意度进一步提升。
　　——各项工作亮点纷呈。我市企业债券发行、金融创新和风险防范等工作受到国务院通报表扬，连续两年享受国家申请企业债券“直通车”政策。获评为全国义务教育教师队伍“县（区）管校聘”管理体制改革示范区、中国特色农产品优势区、全省唯一的全国平安农机示范市、国家中医药健康旅游示范区创建单位、一带一路国际健康旅游目的地、国际健身气功五禽戏之都、全国药膳之都。“互联网+政务服务”“互联网+公积金”等工作在全国推广，开出全省第一张“互联网+自助办税终端”税票，市政府门户网站荣获2017年中国政府网站绩效评估政务公开领先奖、信息公开位居全国302个地市级网站第一名，市中小企业服务中心获批为国家中小企业公共服务示范平台，“金农易贷”荣获全国农村金融十佳品牌创新产品称号，“532”小额贷款保证保险入选中国保险扶贫产品先锋榜，“亳白芍”种植标准化示范区入选第九批国家农业标准化示范项目。市政府连续两年荣获省政府目标管理绩效考核先进单位称号。我市获评为省文明城市、省未成年人思想道德建设工作先进城市、全省创建体育强市示范市，中国好人、安徽好人上榜人数均位居全省第一。济人药业“疏风解毒胶囊二次开发的系统研究及应用”获省科学技术奖一等奖，实现建市以来“零”的突破。涡阳县获评为全国主要农作物全程机械化示范县，农村承包土地经营权抵押贷款试点工作中期评估位居全省第一；蒙城县获评为全国农村职业教育成人教育示范县、全国法治县创建活动先进单位、“中国好粮油”行动示范县；利辛县获评为国家级电子商务进农村综合示范县、全省城乡道路客运一体化示范县，顺利通过国家义务教育发展基本均衡县督导评估认定；谯城区入选全国农民工返乡创业试点，获评为全省“四好农村路”创建示范县（区）、全省农田水利基本建设“江淮杯”一等奖。农业产业化、旅游产业发展等一批省级现场会在我市相继召开。
　　一年来，我们紧紧围绕五大发展理念，重点抓好以下工作：
　　（一）大力推进创新发展，经济增长新动能充分激发。
　　加快现代中医药产业集聚发展。“世界中医药之都”建设迈出坚实步伐，现代中药产业集聚发展基地实现产值321.6亿元、增长23.6%。总投资80亿元的长白山制药（亳州）产业园、11亿元的民生药业产业园、10亿元的美誉医药产业园等39个亿元以上药业项目开工建设。药业工业企业总数达161家，通过GMP认证企业157家。新签约药业工业项目169个、总投资383.1亿元，全国中医药百强企业已有55家落户亳州。现有中成药品种205个，3个品种进入国家医保目录，394个药品技术转移批文上报国家食品药品监管总局待批。成功获批省级中药配方颗粒生产基地，5家企业获批试点生产，5家企业入选中华民族医药百强榜。
　　加快传统产业改造升级。工业“三百工程”和“123工程”扎实推进，完成工业投资389亿元，增长18.3%、居全省第三位；完成工业技改投资136.3亿元，增长19.2%。6家企业入选省2017年度民营企业百强榜。食品制造产业加快发展，新增省级龙头甲级队企业12家，市和县区食品产业园区分别入驻项目46个、56个。规上白酒产业产值突破110.7亿元、增长15.1%，我市荣获“世界十大烈酒名产区”称号。古井集团连续11年入选省百强企业，正宇集团连续5年蝉联全国小麦粉加工企业50强。煤炭开采和洗选业产值增长56.8%，汽车及现代农机装备制造业产值增长7.3%，纺织服装、家具制造、计算机及电子设备制造业产值增长24.9%。
　　加快现代农业提质增效。粮食总产超96亿斤，小麦单产位居全省第一。中药材种植面积发展到116.3万亩，蔬菜种植面积稳定在200万亩。“三品一标”认证农产品达448个，亳丹皮、涡阳大豆获批为国家地理标志农产品。全面推广“一户一块田”，家庭农场发展到5666家，农民专业合作社发展到16905家。新增农业物联网示范点10个。适度规模经营面积420万亩。规模养殖比重达83%。主要农作物耕种收综合机械化率达99%，农机总动力位居全省第一。小型水利工程改造提升项目加快实施，更新改造泵站1235千瓦，加固小型水闸66座，新建、修复机电井7046眼，完成包河、芡河、阜蒙河年度治理任务和淮干一般堤防加固工程。
　　加快现代服务业发展。新增信贷投放300.8亿元，“我要贷款”平台累计发放贷款61.7亿元。新增限上商贸流通企业129家，限上消费品零售额增长16.9%。新业态新模式快速发展，电子商务进农村“两中心、一站点”建设实现全覆盖，筑梦社区、青年创客空间入选国家级众创空间、省级电子商务示范园区，新认定国家级星创天地3家，签约落户服务外包企业10家、7100坐席。邮政业务总量12亿元。大力开展“文化旅游年活动”，完成北关历史街区5条精品线路立面改造，新增市城市展览馆、南湖灯光水舞秀等一批景点，成功引爆全市旅游市场，国内游客、入境游客、旅游外汇收入增速均居全省第一位，旅游总收入、国内旅游收入增速均居全省第二位，城市对外形象和美誉度进一步提升。
　　加快创新创业步伐。新认定高新技术企业20家，高新技术产业增加值增长15.3%，全社会研发经费增长20%，授权发明专利增长66.7%。新增离岸孵化中心、技术转移中心、校企合作联合实验室、省级企业技术中心19家，新建院士工作站3家。新增省级新产品12个、工业精品5个。深入开展“亳州老乡，请您回家”和助力青年学子创业活动，1.2万人返乡创业，投资超500万元创业项目330个、开工建设272个、建成运营173个，带动就业5.1万人，农民工进城就业4.8万人，城镇登记失业率控制在2.9%。
　　（二）大力推进协调发展，统筹联动新格局初步形成。
　　始终坚持规划引领。编制完成市域空间综合规划、全域旅游规划、市区单元规划、公共服务设施规划，城市规划与土地利用总体规划、各专项规划实现有序衔接、有机统一。市中心城区11条道路水系夜景亮化工程建成使用。蒙城县入选全省城市设计（双修）试点县，谯城区十河镇芍花养生小镇获评首批省级特色小镇，古井镇药王村田园综合体获批为全省两个试点项目之一。
　　强力推进城乡建设。积极应对国家融资政策重大调整，引导社会资本参与基础设施、公共服务领域项目建设，落地PPP项目10个、总投资105亿元。实施城建重点项目236个、完工110个，完成投资65亿元；其中，市中心城区实施112个、完工72个，完成投资26亿元。建成高铁片区地下综合管廊3公里，完成海绵城市试点项目3个、黑臭水体治理项目22处。建成棚户区改造住房30043套，改造老城区主次干道雨污分流管网13.2公里、老旧小区22.4万平方米，新建、改建市中心城区环保公厕76个。全面完成美丽乡村建设年度任务。
　　切实加强城乡管理。强化数字城管扁平化指挥系统应用，受理案件61.5万件、结案率97.4%。王合拉垃圾填埋场封场工程开工建设，十河卞铺生活垃圾焚烧发电厂并网发电，城市生活垃圾无害化处理率达99.98%。深入推进农村环境“三大革命”、农村清洁工程、农村垃圾分类处理、集镇集市管理，清理生活垃圾26万吨，收集处置有毒有害垃圾1257吨，完成改厕任务10.3万户，建成污水处理设施95个。
　　（三）大力推进绿色发展，生态宜居新家园加快建设。
　　持续推进“地净、路畅、水活、天蓝、林拥城”工程。突出抓好“五车”数字化监管，实现城区主次干道机械化清洗清扫。市中心城区打通汤王大道等断头路17条，建安路涡河隧道主体工程基本完工，汤王大道涡河隧道加快推进。宋汤河治理五期、龙凤新河、凤尾沟三期等城区水系治理工程全面完工、实现贯通。全面打响“蓝天保卫战”，布设500多个“蓝天卫士”监控探头，秸秆禁烧成效突出。新增人工造林8.8万亩，建设森林生态廊道574公里、生态农田林网95.4万亩，建成“林拥城”绿道60.6公里，创建省级森林城镇8个、森林村庄36个。
　　强化资源节约高效利用。节能减排完成省下达任务，万元GDP能耗下降3%以上。秸秆综合利用率提高到89.9%。建设高效节水灌溉工程6.4万亩，封闭自备井56眼，万元GDP用水量下降8.5%。土地节约集约利用和农村土地综合整治成效突出，新建标准化厂房45.8万平方米，建设高标准农田113万亩，补充耕地3.1万亩，报批建设用地4.3万亩，超额完成永久基本农田划定任务。
　　着力整治环保突出问题。34项中央环境保护督察反馈意见和省突出环境问题整改任务已按规定时限要求销号20项。建立四级河长体系，整治入河排污口152个，西淝河断面水质改善至III类，全市水环境质量同比改善43.6%、居全国276个地级及以上城市第八位。深入开展“全民参与·环保风暴”活动，建成环保督查信息平台及手机APP，关闭或搬迁禁养区内畜禽养殖场185家，新建规模养殖场污染防治设施853家，改造、拆除、关闭砖瓦窑厂137家，改造加油站点地下油罐988个，实施农村环境综合整治项目40个，新增安装使用油烟设施餐饮店2031家，解决了一大批长期遗留的环保突出问题。
　　（四）大力推进开放发展，经济发展新空间持续拓展。
　　狠抓供给侧结构性改革。提前完成煤炭去产能任务，核减煤炭产能150万吨。持续加大土地供应，房地产开发投资增长39%、居全省第三位。认真落实减税降费政策，累计减轻企业负担22亿元。谋划储备项目1651个，总投资3543亿元。新增新三板挂牌企业2家，新增区域股权交易中心挂牌企业81家、其中“现代中医药及健康产业亳州专板”63家，实现直接融资132.2亿元。
　　狠抓重点领域改革。深化“放管服”改革，开展“四送一服”双千工程活动，创优“四最”营商环境。紧盯“只要上一次”目标，改造升级网上办事大厅，8651项入驻服务事项中有8009项实现“一次上网，事就办成”，累计受理申办件914.2万件，办结798.6万件，满意率达99.99%。“我家亳州”APP注册用户突破100万个。“政企直通车”微信平台累计收集企业反映问题4002个，办结3938个。深化商事制度改革，在全省率先颁发“多证合一”营业执照，实现“64证合一”，新增市场主体6.8万户、增量居全省第三位。完成建安集团战略转型，新组建建安投资、城建发展、交通投资、文化旅游控股集团。深化医药卫生体制改革，建立医共体、医联体31个。农村集体产权制度改革和“三变”改革试点扎实推进。公共资源交易全流程电子招投标平台建成运行。户籍管理、城管执法、供销社、国有林场改革全面完成年度任务。
　　狠抓对外开放。新增进出口实绩企业37家、总数达149家；实现进出口总额6.6亿美元，增长27.8%、居全省第三位。实际利用外商直接投资7.8亿美元，增长8%。鼓励引导企业“走出去”，新培育外经主体4家、总数达11家。市政府口岸办正式成立。国家中药材产品质量监督检验中心（安徽）通过国家验收，全国地级市中唯一的中药材技术性贸易措施研究评议基地在我市挂牌运行。成功举办2017年药博会。
　　狠抓精准招商。坚持招大引强，突出工业招商，“立、改、废、释”招商政策，实际利用省外资金530.9亿元、增长34.7%。新落地亿元以上利用省外资金项目192个，其中工业项目147个。市中心城区挂牌出让工业用地176宗、12908亩，分别是上年的2.3倍、3.7倍。坚持引资与引智并重，出台招才引智实施办法，吸引了一批高层次人才来亳工作。
　　狠抓园区转型。6个省级开发区实现生产总值273亿元、财政收入49亿元，分别增长13.2%、31%。亳州经济开发区新开工项目突破100个，财政收入14.1亿元，征收土地2.2万亩，拆迁房屋105.6万平方米，已申报省级高新技术开发区。亳芜现代产业园区获批为省服务外包示范区，谯城经济开发区纳入准国家级经济开发区管理。
　　（五）大力推进共享发展，人民生活新福祉显著增强。
　　精准发力脱贫攻坚。市县区财政共安排专项扶贫资金12.9亿元、是上年的5倍，其中市本级安排8.6亿元、较上年增加7.6亿元。创新开发精准扶贫信息平台，动员1317家单位、58282人参与扶贫帮扶，实现所有贫困村扶贫工作队和帮扶责任人全覆盖。狠抓脱贫攻坚八大工程，整合各类资金投入脱贫攻坚，新增金融扶贫贷款45亿元，推出林业扶贫、文化旅游扶贫等新模式，加强民主监督，实施产业扶贫项目1861个。累计投资32.3亿元，建成并网光伏扶贫电站2201个，总装机规模505.6兆瓦、占全省的25.2%。建成易地扶贫搬迁安置点18个、安置5755人。安置贫困人员就业2.8万人。发放贫困生资助资金4.2亿元，资助学生30.1万人次。发放贫困人口慢性病证9.2万个，享受“两免两降五提高一兜底”政策贫困人口186.2万人次。
　　切实保障和改善民生。城市低保标准提高10%，农村五保集中供养财政补助标准提高28%，农村五保分散供养财政补助标准、低保标准由3150元提升至4310元。实施“关爱工程”，将建档立卡范围外的城乡低保和农村五保对象纳入扶贫政策享受范围。困境儿童保障救助面高于全省平均水平。市养老服务中心、市精神病人福利中心投入使用，23所农村敬老院转型为区域性养老服务中心，建成农村居家养老中心101所。新建、改扩建中小学152所、公办幼儿园41所，亳州学院新增本科专业6个，亳州职业技术学院二期工程开工建设，亳州幼师、亳州中药科技学校二期工程完工，全市职业教育联盟成功组建。华佗中医院新院、市人民医院二期工程建成使用，新建二级民营医院5家。人口自然增长率控制在9‰。送戏下乡演出2592场，送戏进敬老院302场，开展文化广场演出392场，放映公益电影20895场，原创泗州戏《伯俞泣杖》荣获全省十大名剧称号。建成市“五位一体”公共活动中心，建成开放市体育公园、文化公园等4个特色公园，建成投用乡镇（社区）综合文化服务中心5个、农民文化乐园7个、室外标准游泳池10个、城区全民健身路径550套，开工建设市体育馆、市广电中心。成功举办中国（亳州）国际半程马拉松赛、“健康安徽”2017环江淮万人骑行大赛总决赛。
　　着力提升社会治理能力。“七五”普法加快推进。深入实施“排问题、除隐患、喜迎党的十九大”专项行动，社会治理专业化水平大幅提升。扎实开展进京访“百日攻坚”和积案化解专项行动，化解信访积案389件。投资17.5亿元新建视频数据平台高清摄像机4.5万路、总数达5.4万路，全市“两抢一盗”案件发案率下降26.2%。开展安全生产“百日除患铸安”大检查，完成煤矿安全监管职责调整，持续提升消防安全能力，安全生产事故起数、死亡人数和受伤人数全面下降。加强食品药品全环节监管，启动省级食品药品安全城市创建工作。成立市见义勇为基金会。创建全国文明村镇8个、文明单位7个。国防教育、国防动员深入发展，双拥模范城创建工作通过省级验收，人防军事斗争准备工作通过省检验评估验收。妇女儿童、民族宗教、档案、地方志、残疾人、红十字等事业健康发展。统计、外事、侨务、对台、气象、新闻出版、防震减灾、应急管理、无线电管理、机关事务管理和老龄、关心下一代工作取得新成绩。
　　在聚力经济社会发展的同时，我们高度重视政府自身建设。深入推进“两学一做”学习教育常态化制度化，扎实开展“讲政治、重规矩、作表率”专题警示教育，政府系统党的建设全面加强。严格落实“三单六会”和“双调度”制度，完成政府系统年度岗位责任目标任务753项，整改政府工作问题清单事项508项，有效保障了党中央、国务院、省委、省政府和市委各项决策部署的高效落实。自觉接受人大法律监督、工作监督和政协民主监督，市人大代表议案、建议和市政协委员提案办理结果满意率均达99%，法律顾问制度和规范性文件、重大决策事项合法性审查实现全覆盖。创新开设“政风行风热线—市长有约”栏目，在全省率先建成以“12345”市长热线为基础的政府热线统一服务平台。着力强化行政监察和审计监督，纵深推进党风廉政建设和反腐败斗争，政府系统的执行力和公信力进一步提升。
　　所有这些成绩的取得，是省委、省政府和市委正确领导的结果，是市人大和市政协监督支持的结果，是全市646万人民团结一心、苦干实干的结果。在此，我代表市人民政府，向在各个岗位上辛勤工作的全市人民，向给予政府工作大力支持的全体人大代表、政协委员，向各民主党派、工商联、各人民团体和社会各界人士，向驻亳解放军指战员、武警官兵和政法干警，致以崇高的敬意！向所有参与亳州建设的各位投资者和关心支持亳州发展的各界朋友，表示衷心的感谢！
　　各位代表！回首2017年，挑战无处不在，历程充满艰辛，成绩来之不易。我们深深体会到：唯有不忘初心、牢记使命，坚持党的领导，紧紧依靠全市人民，才能办好亳州的事情；唯有坚持以人民为中心的发展思想，维护群众利益，不断满足人民日益增长的美好生活需要，才能赢得信任和支持；唯有忠诚担当、埋头苦干，努力比别人干得更多、干得更实、干得更拼，才能实现跨越赶超、走在前列。
　　同时，我们也清醒地看到，发展中还存在不少问题、面临不少挑战：经济总量不大，产业结构不优，质量效益不高，创新能力不强，现代服务业发展不快，文化旅游开发深度不够；脱贫攻坚压力巨大，乡村振兴任务艰巨，重点领域改革需要持续深化，基础设施建设存在不少短板，污染防治任重道远，人民日益增长的美好生活需要和不平衡不充分的发展之间的矛盾较为突出；少数部门争先进位意识不强，少数干部懒政怠政行为依然存在。对此，我们要以对党、对人民高度负责的精神，直面问题，认真加以解决。

****二、2018年目标任务****

　　2018年是贯彻党的十九大精神的开局之年，是改革开放40周年，是决胜全面建成小康社会、实施“十三五”规划承上启下的关键一年，做好政府工作意义重大。纵观当前形势，挑战与机遇并存。党中央、国务院大力实施中部崛起战略、健康中国战略，强力推进中医药和大健康产业发展。省委、省政府大力实施皖北振兴战略，加快淮河生态经济带建设，支持我市打造“世界中医药之都”。这些都为亳州借势崛起、弯道超车提供了难得的历史机遇和广阔的发展空间，我们有信心、有条件、有能力把握机遇、乘势而上，推动新时代亳州实现高质量发展。
　　今年经济社会发展的指导思想是：全面贯彻落实党的十九大精神和中央经济工作会议精神，以习近平新时代中国特色社会主义思想为指导，深入贯彻落实习近平总书记视察安徽重要讲话精神，紧扣社会主要矛盾变化，按照高质量发展的要求，坚持党对经济工作的领导，坚持稳中求进工作总基调，按照“深入践行五大发展理念，奋力走在皖北振兴前列”工作要求，统筹推进“五位一体”总体布局和协调推进“四个全面”战略布局，以供给侧结构性改革为主线，以五大发展行动计划为抓手，着力做好稳增长、促改革、调结构、惠民生、防风险各项工作，大力推进改革开放，大力推进创新驱动，大力推动质量变革、效率变革、动力变革，全力打好防范化解重大风险、精准脱贫、污染防治攻坚战，努力实现更高质量、更有效率、更加公平、更可持续的发展，全面开创现代化五大发展美好亳州建设新局面。
　　今年经济社会发展的主要预期目标是：地区生产总值增长8.3%，财政收入增长8.3%，固定资产投资增长10.5%，规模工业增加值增长8.6%，战略性新兴产业产值增长18%，社会消费品零售总额增长10%左右，进出口总额增速高于全省平均水平，城镇常住居民人均可支配收入增长8%左右，农村常住居民人均可支配收入增长8.6%左右，节能减排、大气和水污染防治完成省政府下达的目标任务。
　　围绕实现上述目标，重点做好以下五个方面的工作：
　　（一）强力深化供给侧结构性改革，加快建设特色鲜明、优势突出的新兴工业强市。坚持把提高供给体系质量作为主攻方向，聚焦优势产业，实施创新驱动战略，推动实体经济发展，加快培育新动能、升级传统动能、激发开发区活力，力争新增规模工业企业100家。
　　一是强主导完善产业链条。坚持现代中医药产业主导地位，紧盯建设“世界中医药之都”目标，编制完成《“世界中医药之都”发展规划》，谋划设立中医药产业发展母基金，加快推进现代中药产业集聚发展基地建设。延伸现代中医药产业链条，完成中医药产业固定资产投资120亿元、增长20%，实现中医药工业产值350亿元、增长20%，中药材种植产值35亿元、增长10%，流通业贸易额500亿元、增长25%，力争产业规模突破1000亿元。加大药品品种转移引进力度，完成药品技术转让或委托加工受理协议品种200个以上。推进省级中药配方颗粒基地建设，指导5家试点企业各申报备案品种100个以上，启动第二批试点企业申报工作。引导5家企业开展中药破壁饮片生产试点。加大政策扶持力度，促进医疗器械产业发展。
　　二是强基础补齐工业短板。围绕五大发展行动计划，谋划重点项目1200个以上，总投资3600亿元以上。继续坚持“四督四保”制度，强力推进700个以上省市重点项目建设，完成年度投资900亿元以上。充分发挥重大项目牵引作用，建成美誉医药产业园、群康药业、汉盛中成药、中建材泰山石膏板材、中嘉铝合金模板、婉悦纺织等一批亿元以上重大产业项目，开工建设华能风电、信湖煤矿、板集煤矿复建、中青健康中药大数据产业园、梧桐树纺织产业园等一批亿元以上重大产业项目，争取获得涡北低热值煤发电新建项目核准立项。加快建设太和至亳州天然气干线。建成投运220千伏华佗输变电工程，开工建设220千伏乐土输变电工程。
　　三是强技术加快创新发展。坚持把创新作为引领发展的第一动力，强化企业研发激励，设立现代中医药成果转化基金，培育省级企业技术中心5家、新产品6个，申报省级及以上重大科技项目20项，新认定高新技术企业15家，实现高新技术产业增加值增长12%。继续实施工业“三百工程”和“123工程”，推动白酒酿造、煤炭及煤化工、纺织服装、建筑材料等传统产业改造升级，力争工业投资增长13%、技改投资增长15%，着力培育一批具有创新能力的排头兵企业。加大招才引智力度，重点引进高层次科技人才团队，推动产学研深度对接，新创建重点实验室、新型研发机构、技术创新中心、技术转移中心、离岸孵化器等高水平创新平台5家以上。促进企业家素质“提升”、高技能人才队伍“提量”、本土型人才技术“提优”，培养高技能人才2000人，新建技能大师工作室4个。强化品牌建设，培育申报中国驰名商标1件，新增省著名商标15件、名牌产品6个。
　　四是强平台推动集聚发展。坚持把开发区作为经济发展的主战场，瞄准产业链中高端，大力发展新一代信息技术、新能源汽车、大数据、高端装备制造、新型建材等产业，加快建设浪潮（亳州）大数据产业园等特色园中园，确保6个省级开发区生产总值超300亿元、固定资产投资超600亿元、财政收入超60亿元。加快开发区改革和创新步伐，支持谯城经济开发区创建国家级经济开发区、亳州经济开发区创建省级高新技术开发区，推进三县开发区特色化、差异化发展。
　　五是强环境激发市场主体活力。持续创优“四最”营商环境，常态化推进“四送一服”双千工程活动，用好“政企直通车”微信平台，穷尽办法、倾尽所能、依法依规帮助企业解决实际问题，着力激发和保护企业家精神，加快构建亲清新型政商关系，充分释放创新创业活力。深化国企国资改革，支持古井集团和建安投资、城建发展、交通投资、文化旅游控股集团做强做优做大。实施民营企业扶持计划和中小企业培育计划，落实减税降费优惠政策，清理规范涉企保证金，提高金融支持力度，降低企业各类成本，激发企业内生动力，新增个体工商户2.5万户、私营企业1万户，确保民营经济增加值增长9%以上。持续推进“亳州老乡，请您回家”和助力青年学子创业活动，推动筑梦社区、青年创客空间争创国家级创业创新示范基地，让亳州老乡和青年学子愉快创业。
　　（二）强力实施乡村振兴战略，加快建设绿色高效、优质安全的现代农业强市。坚持把“三农”工作作为重中之重，大力实施乡村振兴战略，培养造就一支懂农业、爱农村、爱农民的“三农”工作队伍，让农业成为有奔头的产业、农民成为有吸引力的职业、农村成为安居乐业的美丽家园。
　　一是坚决打赢脱贫攻坚战。坚持精准扶贫、精准脱贫，把提高脱贫质量放在首位，尽锐出战，精准施策，巩固谯城区、蒙城县脱贫成果，扎实做好利辛县、涡阳县摘帽验收工作。统筹推进脱贫攻坚八大工程，每个贫困村发展1项以上特色种养业，40%的贫困村建成“一村一品”专业村，落实贫困人口“三保障一兜底一补充”政策，抓好建档立卡贫困户人均1亩生态林建设，改造农村危房10690户，光伏扶贫电站发电6亿千瓦时，强化易地搬迁扶贫户后续帮扶，推进贫困户零就业家庭动态清零，探索建档立卡贫困学生高中招生降分录取，稳步推进金融扶贫、社保兜底脱贫。坚持脱贫不脱责任、脱贫不脱政策、脱贫不脱帮扶、脱贫不脱监管，持续完善脱贫户后续扶持政策，着力构建防范返贫长效机制，确保脱贫成果经得起历史和人民群众的检验。
　　二是持续深化农村综合改革。保持土地承包关系稳定并长久不变，第二轮土地承包到期后再延长30年。稳妥推进农村集体产权制度改革试点，全面完成农村集体资产清产核资任务。每个乡镇选择1个村开展农村“三变”改革试点。深化农业供给侧结构性改革，建设粮食绿色增产创建示范点60个、优质专用粮食生产基地200万亩，新增中药材标准化种植面积2万亩、设施蔬菜水果5万亩、市级以上蔬菜水果标准园10个，创建绿色循环标准化养殖示范场60个。认真落实粮食安全行政首长负责制，全社会粮食收购130万吨，新增“放心粮油”示范店、“主食厨房”直营店各20家。完成县区供销社改革。
　　三是大力发展现代农业。加快构建现代农业产业体系，推进质量兴农、绿色强农，促进粮经饲统筹、农林牧渔结合、种养加一体、一二三产融合发展，新增“三品一标”农产品61个，新认定市级以上现代农业示范园区60个，新增规模以上农产品加工企业20家，每个县区食品产业园新入驻企业5家，力争全市产值超亿元龙头企业达125家，规模以上农产品加工产值增长11%。加快构建现代农业生产体系，大力提升农业机械化水平，新增部、省级农机专业合作示范社8家以上，农机作业托管面积达90%以上。加快构建现代农业经营体系，大力培育新型职业农民和新型经营主体，新建、改造农家店150家、农村综合服务社（中心）25家，新增省级示范农民专业合作社6家、示范家庭农场12家，土地适度规模经营面积达440万亩。完善农业保险服务体系。
　　四是加快建设美丽乡村。全面完成美丽乡村建设年度任务，确保实现“皖北居前列、全省争先进”目标。持续推进基础设施和公共服务向农村延伸，启动天然气镇镇通工程，完成农村电网改造升级投资2亿元，新建、改建农村危桥152座，建成非建档立卡村通村硬化道路500公里，打造一批“四好农村路”。深入实施农村清洁工程，继续推进农村环境“三大革命”和垃圾分类处理，完成农村环境综合整治任务40个，改造农村厕所10万座，启动80个乡镇政府驻地生活污水处理设施建设、建成率达80%。深化乡风文明建设，推动移风易俗，全面建立红白理事会。
　　五是着力拓宽农民增收渠道。扎实推进农村电商全覆盖，培育电子商务特色小镇12个，建设电商示范村40个，发展“互联网+农村超市”600家，农产品电子商务销售额突破15亿元。大力实施旅游富民工程，推动“百村景区化”，打造乡村旅游精品线路4条，创建省级优秀旅游乡镇2个、省级旅游示范村3个、3星级以上农家乐10家。加快推进引江济淮亳州段工程，开工建设十河闸、高阁闸、贾集闸除险加固工程，完成阜蒙河、芡河利辛段、包河治理工程，启动北淝河涡阳段、芡河蒙城段、油河治理工程，完成高效节水灌溉工程5.1万亩。
　　（三）强力扩大双向开放，加快建设底蕴厚重、功能完善的文化旅游强市。坚持招商引资第一要事不动摇，持续推动开放大提速，加快现代服务业大发展，继续开展文化旅游年活动，把文化旅游业培育成我市经济发展的重要支柱产业和新的增长极。
　　一是扎实推进精准招商。持续开展“千家知名企业招商攻坚”活动，实际利用省外资金增长10%以上。坚持工业项目为主、药业项目为先、亿元项目为重，突出现代中医药、食品制造、文化旅游、服务外包、战略性新兴产业等，充分发挥亳州之友联谊会、亳州商会作用，大力开展精准招商、驻点招商、借会招商、以商招商、委托招商、平台招商，力争签约亿元以上工业项目150个，每个招商载体至少引进5亿元以上项目5个、10亿元以上项目2个。坚持把项目开工建设作为考核认定条件，新建亿元以上利用省外资金项目150个，确保项目引进来、落下地、见成效。
　　二是持续扩大对外开放。积极承接发达地区产业转移，完善奖励政策，提升外商直接投资吸引力。主动参与“一带一路”建设，引导有条件的企业在境外设立营销机构及服务网络，持续拓展海外市场。加强对外开放窗口平台建设和口岸管理，完善国检和海关机构，探索设立“无水港”。实施外贸主体培育工程，孵化新增进出口企业25家以上。采取社会化模式办好新一届药博会，打造世界知名中医药展会品牌。
　　三是大力发展文化旅游业。紧盯全域旅游目的地建设，以国家中医药健康旅游示范区创建为抓手，落实《全域旅游发展规划》，开展旅游新兴业态培育、精品线路整合、旅游商品开发、景区管理提升、公共服务提升、营销平台建设等专项行动，举办2018亳州文化旅游年活动，全年接待国内游客2300万人次，实现旅游总收入190亿元。实施精品示范工程，开工建设中华中医药博览园、百花园、陈抟文化小镇、古井白酒文化特色小镇等重大旅游项目，加快推进亳药花海休闲观光大世界项目二期建设和北关历史街区改造升级，深入推进亳州古城旅游区、古井旅游区创建国家5A级旅游景区和林拥城景区创建国家4A级旅游景区工作，创建国家3A级以上旅游景区3家。实施服务提升工程，启动实施智慧旅游项目，完善旅游标识系统，建设南北游客集散服务中心，加快花戏楼名吃一条街等特色旅游商业街改造，规划建设旅游综合体10个，新建、改扩建旅游厕所200座。实施市县联动工程，以“道源问道”研学游为主线，培育特色旅游小镇，评选亳州旅游“十大名片”，推进道源国家湿地公园、庄子祠景区改造提升、西淝河国家湿地公园和古井乐酒家园等项目建设，串珠成线、连线成面，持续打造全域旅游品牌。
　　四是加快发展现代服务业。坚持金融服务实体经济导向，新增金融机构网点5家，投放信贷资金180亿元；着力完善多层次资本市场，力争新增IPO报备企业1家、新三板挂牌企业2家、区域股权托管交易中心挂牌企业20家，支持“现代中医药及健康产业亳州专板”企业转板，实现直接融资50亿元；稳妥处置地方政府债务，着力化解金融风险，坚决打好防范化解重大风险攻坚战。积极培育健康养生、共享经济、数字经济等新业态新模式，大力推进医养融合发展，完成公建民营养老机构改革试点8所，新增社会办养老床位500张、居家养老服务站点12所。新增限上商贸流通企业90家。加快发展交通运输、邮电通讯和商贸餐饮业，确保邮政业务总量15亿元。
　　（四）强力提升城市能级，加快建设互通互联、方便快捷的新型智慧城市。围绕“省际交汇区域中心城市”定位，以新型智慧城市建设为统领，以“四创一建”为抓手，着力完善城市功能、健全交通网络、美化城市环境、优化政务服务，加快构建市县一体、互融共通、多点支撑的发展格局。
　　一是提升城市承载能力。持续开展“九城同创”，大力开展“两治三改”，积极创建全国文明城市。坚持规划先行，建成空间规划信息平台，实现规划方案及施工图电子报批，统筹做好城市“双修”工作。计划投资181.7亿元，在市中心城区实施165个道路、桥梁、供排水、停车场、污水和餐厨废弃物处理项目。实施PPP项目24个、总投资440亿元。持续提升物业管理水平，实现物业管理新建小区全覆盖、老旧小区覆盖80%以上。
　　二是推动县域振兴发展。加快培育县区主导产业，支持涡阳县重点发展光机电、绿色装配式建筑、循环经济等产业，蒙城县重点发展新能源汽车及汽车电子、生物质发电、新型建材及智能家居等产业，利辛县重点发展煤电煤化工、丝网服饰、光伏等产业，谯城区重点发展现代中医药、白酒等产业，建设一批产学研和科技成果转化平台，创建一批特色产业集聚发展基地。坚持区域联动、功能互补，支持三县加快推进新城区路网、电网、水网、绿化、亮化等项目建设，加快实施老旧小区改造、供水管网改造、断头路打通等工程，持续完善停车场、健身广场、街头游园、文体场馆等公共服务，实现新城展新貌、老城换新颜。高品质建设一批特色小镇。
　　三是打造互联互通交通网络。大力实施对外交通畅通工程，加快建设商合杭高铁、亳州高铁南站和站前广场、涡河高等级航道整治等项目，完成亳州机场立项和可研、专题编制，新建、续建国省干线公路190公里。大力实施市域交通畅通工程，加快亳蚌城际铁路、淮阜城际铁路、亳蒙高速、涡阳船闸、涡河蒙城枢纽前期工作。大力实施市内交通畅通工程，建成亳州北公交客运枢纽站，加快建设高铁公路客运枢纽站和杜仲路等6条下穿铁路立交桥，加快推进高铁片区综合管廊、中心城区快速路网一期、三清大道及涡河大桥、幸福桥改造等99项路桥工程。
　　四是深入推进“地净、路畅、水活、天蓝、林拥城”工程。强化“五车”监管，推广使用全封闭环保建筑垃圾运输车。完善药王大道、三圣路、建安北路等中心城区12条道路建设，建成建安路涡河隧道，完成汤王大道涡河隧道主体工程。建成大中型涵闸远程监控系统二期工程，完成凤尾沟三期、龙凤新河和团结沟主体工程。坚决打好“蓝天保卫战”，重点做好扬尘污染防治、燃煤锅炉和砖瓦窑厂治理等工作，确保主要污染物排放总量持续下降、空气环境质量持续改善。全面落实河长制，建立林长制，扎实开展国土绿化行动，新增人工造林10万亩，创建省级森林城镇4个、森林村庄27个，实现林拥城全面贯通。
　　五是建设新型智慧城市。深化“放管服”改革，清理规范涉企服务性收费，动态调整权责清单、公共服务清单、中介服务清单，公布2018年清单目录。紧盯“只要上一次”目标，建成网上办事大厅二期工程，实现96%以上的为民服务事项“一次上网，事就办成”，打造“互联网+政务服务”升级样板。深入推进数字城管建设，实现市县一体、互联互通。完成市视频数据平台机房扩容和区域补盲工作，持续拓展视频数据应用范围。深化国地税联合办税和社会综合治税，推进国地税“一窗业务统办”，推广“互联网+办税自助终端”应用。持续深化商事制度改革，认真做好涉企信息归集和“双随机、一公开”工作。实施“互联网+招标采购”，确保符合条件的项目全流程电子招投标和网上商城采购比率均达90%。整合升级以“12345”市长热线为基础的政府热线统一服务平台，全天候、多方位、高效率服务群众和企业。
　　（五）强力保障改善民生，加快建设生态宜居、文明和谐的健康养生城市。坚持以人民为中心的发展思想，着力保障人民群众基本生活，完善公共服务体系，优化生态人居环境，促进社会公平正义，让人民群众的获得感、幸福感、安全感更加充实、更有保障、更可持续。
　　一是切实提高保障和改善民生水平。全面完成省政府下达的民生工程任务。持续抓好就业工作，新增城镇就业3.2万人，转移农村劳动力7.2万人，进城就业农民工2.1万人，城镇登记失业率控制在4%以内。拓展社会保障卡应用功能，推进城乡居民基本医疗保险整合，确保新农合参合率95%以上、住院补偿比70%以上。坚持“房子是用来住的、不是用来炒的”定位，加强分类调控，保持房地产市场健康发展。投资89.6亿元，新开工棚户区改造住房16299套。大力加强基层民政能力建设，继续实施“关爱工程”，城市、农村低保标准分别提高5%和8%。深入实施农村饮水安全巩固提升工程。做好城乡困难群体法律援助工作。加大农村留守儿童和妇女、老人、残疾人关爱力度。
　　二是切实扩大优质公共服务供给。办好公平优质教育，加大财政投入，改善提升办学条件，推动城乡义务教育由基本均衡向优质均衡转变。深化中小学幼儿园教师无校籍管理改革，出台优秀教师引进和奖励办法，实施乡村教师支持计划、薄弱学校改造工程和教育教学质量提升工程，加快乡村学校内涵建设和校园信息化建设，新建、改扩建中小学46所、公办幼儿园35所。支持亳州学院新增3—5个本科专业、开工建设四期工程，加快亳州职业技术学院二期工程建设，力争引进省内外知名中医药大学在我市建设分校（校区）。推进“健康亳州”建设，深化公立医院编制周转池试点，完善现代医院管理制度，健全县域医共体机制，推行分级诊疗和家庭医生签约服务，进一步提升基层卫生服务能力。加快建设市第二人民医院、市妇幼保健计划生育服务中心，开工建设二级及以上民营医院3家。争创全国基层中医药工作先进市，推进华佗中医院创建“三甲中医院”。全年人口自然增长率控制在9.5‰以内。深入开展文化惠民活动，加快市广电中心建设，建成市文化馆和5个全民阅读基层示范点，建设贫困村综合文化服务中心50个，举办广场公益文艺演出300场，提升全民科学文化素质。认真落实《亳州国家历史文化名城保护条例》，加强非物质文化遗产传承与保护，成立涡河文明研究中心和市文物保护基地，推进五禽戏申报世界非物质文化遗产工作，组织申报第八批全国重点文物保护单位。推进体育强市创建，建成市体育馆，完成市体育场改造，建设社区文体中心15个以上、小型便民利民健身场地100块以上，举办国际全程马拉松等知名赛事。
　　三是切实打好污染防治攻坚战。全力整改中央环保督察反馈意见和省突出环境问题，深入开展“全民参与·环保风暴”活动，确保按期完成整改任务。坚决打好碧水攻坚战，深入实施“水十条”，开展农村黑臭水体排查治理，完成城市黑臭水体治理9处，整治入河排污口98个，西淝河马店孜断面保持Ⅲ类水质，涡河义门大桥、岳坊大桥断面保持V类水质，涡河龙亢断面保持Ⅳ类水质。坚决打好净土持久战，全面实施“土十条”，开展农用地土壤污染状况详查，实现主要农作物化肥和农药使用量零增长、规模养殖场治污设施全覆盖。推进生态环境气象保障工程建设，新建亳蒙一级公路能见度监测点10个。拓展生态保障制度覆盖面，开征环境保护税，推进环保机构监测监察执法垂直管理制度改革，严格执行生态环境损害责任终身追究制度。
　　四是切实高效节约利用资源。坚决落实最严格的水资源管理制度，用水总量控制在11.2亿立方米，万元GDP用水量下降6.2%。坚持节约集约用地，严格工业用地标准，加快标准化厂房建设，推进低效建设用地再开发，提高土地利用效益。坚决落实最严格的耕地保护制度，严查违法违规用地案件，加强农村土地综合整治，完成建设用地增减挂钩1.6万亩、工矿废弃地复垦利用3000亩、补充耕地2万亩，实施高标准农田70万亩以上。推进县区秸秆综合利用产业园建设，建成上海电气蒙城生物质发电项目、凯迪利辛生物质秸秆发电厂，完成凯迪谯城生物质秸秆发电厂主体工程，加快建设光大集团涡阳生物质发电项目，开工建设润盟环保秸秆板材项目，农作物秸秆综合利用率达90%以上。
　　五是切实加强和创新社会治理。深化安全生产专项治理和打非治违专项行动，推进基层安全生产网格化全覆盖，构建安全风险分级管控和隐患排查治理双重预防机制，排查一般隐患1万处以上、重大隐患180处以上，整改率98%以上。坚持食品药品安全“党政同责”，按照“四个最严”要求，加大食品、药品、医疗器械等生产经营企业飞检力度，推进创建省级食品药品安全城市。深入开展信访基层基础业务规范化建设年和信访积案化解专项行动，打造阳光信访、责任信访、法治信访。健全社会信用体系，加快“信用亳州”建设。完善社会治安防控体系，深化“一村一警”工作，严密防范和依法打击各类违法犯罪行为，着力提高社会治理水平，全力构建“平安和谐亳州”。大力弘扬社会主义核心价值观，继续开展道德模范、身边好人和寻找“最美家庭”评选、推荐、宣传活动，全面提升公民文明素质和道德水平。加强全民国防教育、国防动员和后备力量，建设军民融合产业园，推进军民深度融合发展。健全完善消防体系，推动消防事业发展。深化统计管理体制改革，开展第四次全国经济普查。完成“七五”普法中期考核验收工作。做好第十届村委会和第六届社区居委会换届选举工作。建成市人防基本指挥所。大力推进妇女儿童、民族宗教、档案、地方志、红十字、外事、侨务、对台、新闻出版、防震减灾、应急管理、公积金管理、无线电管理、机关事务管理和老龄、关心下一代等事业发展。

****三、加强政府自身建设****

　　新时代要有新气象，更要有新作为。我们不能有任何喘口气、歇歇脚的念头，要始终强化“每天都是节点、全年都在冲刺”的意识，以“首战用我、用我必胜”的担当，以“见红旗就扛、见第一就争”的勇气，以“一日不为、三日难安”的紧迫感，夙夜在公，连续作战，全身心投入，苦干实干拼命干，用我们的辛苦指数，换取亳州经济社会发展的质量指数和速度指数，换取亳州人民的幸福指数和满意指数，不断满足人民日益增长的美好生活需要。
　　（一）坚定信仰，对党忠诚。旗帜鲜明讲政治，严守政治纪律和政治规矩，始终在政治立场、政治方向、政治原则、政治道路上同以习近平同志为核心的党中央保持高度一致。持之以恒强党性，深入开展“不忘初心、牢记使命”主题教育，持续强化政治能力，全面提高“八种本领”，切实增强对党忠诚、为党分忧、为党尽职、为民造福的政治担当，永葆共产党人的政治本色。矢志不渝跟党走，强化“四个意识”，增强“四个自信”，坚决维护党中央的权威，坚决服从党中央的集中统一领导，坚决用习近平新时代中国特色社会主义思想武装头脑、指导实践、推进工作。
　　（二）不忘初心，执政为民。始终坚持以人民为中心的发展思想，把人民放在心中最高的位置，切实解决环境治理、“看病难、看病贵”“择校热”“大班额”“入托难”、结构性就业矛盾等群众最关心最直接最现实的利益问题，让发展更有温度，让幸福更具质感。着力畅通为民服务渠道，办好“12345”市长热线、“政风行风热线—市长有约”“沟通—政务零距离”，健全完善问题交办销号机制，确保事事有着落、件件有回音。坚持无禁区、全覆盖、零容忍，严查发生在群众身边的不正之风和腐败行为，保持反腐败斗争压倒性态势。持续加强政务诚信，强化政务公开，着力保障人民群众的知情权、参与权、选择权和监督权，不断增强政府的公信力和执行力。
　　（三）勇立潮头，敢为人先。始终保持忧患意识，清醒认识到原地踏步就是退步，发展慢了也是退步，坚决抛弃小富即安、小进则满、松懈疲沓思想，瞄准目标、提升标杆，找准短板、开准药方，定向施策、久久为功。切实增强担当精神，严格执行“三单六会”和“双调度”制度，聚焦脱贫攻坚、环境保护、安全生产、招商引资、项目建设等重点工作，扭住目标不放，盯住问题不松，敢于啃硬骨头、敢于涉险滩，雷厉风行、紧抓快办。牢固树立实干作风，坚持“四化、三可”和“快、好、高”标准，对确定的目标、制定的措施、部署的任务，下深功夫、下苦功夫、下硬功夫抓好落实，继续打造一批亳州亮点、创造一批亳州经验。
　　（四）依法行政，廉洁从政。全力推进法治政府建设，坚持法无授权不可为、法定职责必须为，认真执行重大行政决策公众参与、专家论证、风险评估、合法性审查、集体讨论决定等程序，健全完善政府立法体制机制，加强财政预算管理，及时公开预决算和“三公”经费信息，持续强化审计监督。自觉接受人大法律监督、工作监督和政协民主监督，主动接受社会监督、舆论监督，认真办理人大代表议案建议和政协委员提案，组织实施年度协商计划，广泛听取各民主党派、工商联、无党派人士意见建议，充分发挥人民团体的重要作用。坚持廉洁从政，自觉遵守党章党规，严格执行中央八项规定精神及省、市相关规定，严防“四风”反弹，着力打造忠诚、干净、担当的干部队伍。
　　各位代表！使命呼唤担当，使命引领未来。让我们在市委的坚强领导下，团结带领全市人民，不忘初心，牢记使命，奋发有为，真抓实干，奋力谱写新时代中国特色社会主义的亳州篇章！

****政府工作报告名词解释****

　　1.四个导向——即问题导向、目标导向、服务导向、务实导向。
　　2.四个点问题——即人民群众关心关注的热点问题、社会各界反映强烈的焦点问题、当前工作中亟待解决的重点问题、制约亳州长远发展的难点问题。
　　3.“532”小额贷款保证保险——指为满足小型微型企业、农村种植养殖大户和城乡创业者等生产经营活动中的小额融资需求，由借款人投保小额贷款保证保险，银行以此作为主要担保方式，向借款人发放贷款的一种业务模式，一旦发生代偿，保险公司、政府与银行按5:3:2的比例承担贷款本息损失。
　　4.四好农村路——即建好、管好、护好、运营好农村公路。
　　5.工业“三百工程”——即实施100个在建项目、100个亿元项目、100个技改项目。
　　6.工业“123工程”——即培育100家小巨人企业、200家成长性小企业、300家基础较好的小微企业。
　　7.三品一标——即无公害农产品、绿色食品、有机食品、农产品地理标志。
　　8.两中心、一站点——即县级电子商务公共服务中心、农村电子商务物流配送中心、乡村电子商务服务网点。
　　9.农村环境“三大革命”——即农村垃圾、污水、厕所专项整治。
　　10.五车——即洒水车、清扫车、垃圾运输车、渣土车、混凝土搅拌车。
　　11.新三板——即全国中小企业股份转让系统，是经国务院批准，依据证券法设立的继上海证券交易所、深圳证券交易所之后第三家全国性证券交易场所，主要为全国创新型、创业型、成长型非上市中小微企业发展服务。
　　12.城市双修——即生态修复、城市修补，是治理“城市病”、改善人居环境、转变城市发展方式的有效手段。
　　13.PPP模式——即公共私营合作制，指政府与营利性、非营利性企业，为合作建设城市基础设施项目或提供某种公共物品和服务，以特许权协议为基础，彼此之间形成一种伙伴式的合作关系，并通过签署合同来明确双方的权利和义务，以确保合作的顺利完成，最终使合作各方达到比预期单独行动更为有利的结果。
　　14.四送一服——即送发展理念、送支持政策、送创新项目、送生产要素，服务实体经济。
　　15.四最——即审批事项最少、办事效率最高、投资环境最优、市场主体和人民群众获得感最强。
　　16.三变——即资源变资产、资金变股金、农民变股东。
　　17.两免两降五提高一兜底——“两免”即：免除贫困人口新农合个人缴费部分，免除住院预付金。“两降”即：降低贫困人口住院治疗的补偿起付线，降低贫困人口大病保险起付线。“五提高”即：提高贫困人口普通门诊医药费用限额内实际报销比例，提高常见慢性病门诊实际报销比例，提高按病种付费补偿比例，提高大病保险分段补偿比例，提高贫困人口医疗救助水平。“一兜底”即：贫困人口通过综合补偿后，剩余部分合规医药费用实行政府兜底保障。
　　18.三单六会——“三单”即：政府系统年度岗位责任目标任务清单、政府工作问题清单、市长工作提示单。“六会”即：市政府常务会、市长工作例会、市长专题调度会、市政府双调度会、市规委会、市土委会。
　　19.双调度——即市政府对经济工作、项目建设、环境保护和安全生产，实行单月分管副市长调度，双月市长和分管副市长共同调度的工作机制。
　　20.四督四保——即督查推进新建项目，保证开工率；督查推进续建项目，保证竣工率；督查推进竣工项目，保证达产率；督查推进储备项目，保证转化率。
　　21.三保障一兜底一补充——“三保障”即：提高基本医保、大病保险、医疗救助保障待遇水平。“一兜底”即：设定政府兜底保障线。“一补充”即：实行慢性病门诊补充医疗保障。
　　22.无水港——即在内陆地区建立的具有报关、报验、签发提单等港口服务功能的物流中心。
　　23.IPO——即首次公开募股，指一家企业或公司（股份有限公司）第一次将它的股份向公众出售。
　　24.四创一建——即创新城市发展方式、创建城市特色风貌、创造城市优良环境、创优城市管理服务，建设绿色江淮美好家园。
　　25.两治三改——即治理违法建设，治理违法用地；推进城镇棚户区改造，实施城中村改造，推进城市老旧小区综合整治、危房和非成套住房改造。
　　26.双随机、一公开——即检查对象、执法检查人员随机抽取产生，检查情况和查处结果及时向社会公开。
　　27.四个最严——即最严谨的标准、最严格的监管、最严厉的处罚、最严肃的问责。
　　28.八种本领——即学习本领、政治领导本领、改革创新本领、科学发展本领、依法执政本领、群众工作本领、狠抓落实本领、驾驭风险本领。
　　29.四化、三可——即实化、细化、量化、具体化，可比较、可检验、可考核。
　　30.快、好、高——即工作节奏和发展速度要快、工作成果和质量效益要好、在全省和皖北位次要高。
